# Unnecessary Antibiotics in Older Female Patients with Recurrent Urinary Tract Infections

**DOI:** 10.1007/s00192-025-06141-x

**Published:** 2025-04-29

**Authors:** Elizabeth Critchlow, Alexandra Kuzma, Nathanael Koelper, Surbhi Agrawal, Lauren Dutcher, Lily Arya

**Affiliations:** 1 Department of Obstetrics and Gynecology, University of Pennsylvania, Philadelphia, PA USA; 2 Department of Obstetrics and Gynecology, Crozer Health, Upland, PA USA; 3 Women’s Health Clinical Research Program, Department of Obstetrics and Gynecology, University of Pennsylvania, Philadelphia, PA USA; 4 Division of Urogynecology, Department of Obstetrics and Gynecology, University of Pennsylvania, Philadelphia, PA USA; 5 Division of Infectious Diseases, Department of Medicine, University of Pennsylvania, Philadelphia, PA USA

**Keywords:** Antibiotic stewardship, Antibiotic resistance, Urinary tract infection, Antibiotic prescriptions, Social vulnerability, Asymptomatic bacteriuria

## Abstract

**Introduction:**

Our aim was to describe the rate of unnecessary antibiotic prescriptions across specialties and the frequency of clinical scenarios in which the unnecessary antibiotics were prescribed in older female patients with recurrent urinary tract infections (UTI).

**Methods:**

This was a retrospective cohort study of female patients 65 or older with a clinical diagnosis of recurrent UTI. An unnecessary antibiotic was defined as an antibiotic prescribed (i) for asymptomatic bacteriuria (positive culture in the absence of UTI-specific symptoms), (ii) in the absence of UTI-specific symptoms, or (iii) in the presence of documented negative urine culture or negative pyuria. Data on clinical scenarios during episodes when an unnecessary antibiotic was prescribed (such as symptom documentation, urine testing) were extracted and described.

**Results:**

The overall rate of unnecessary antibiotics across 454 episodes of antibiotic prescriptions in 175 older female patients with recurrent UTI was 41% and did not significantly differ between specialties (primary care 45%, urogynecology 41%, obstetrics-gynecology 29%, urology 28%, urgent care 27%, *p* = 0.06). The commonest clinical scenario during which an unnecessary antibiotic was prescribed was absence of documented UTI-specific symptoms (60%) followed by asymptomatic bacteriuria (46%). Other clinical scenarios associated with unnecessary antibiotics included antibiotics prescribed with documented negative pyuria (32%) or negative urine culture (18%). In 11% of episodes, antibiotics were prescribed without any documented UTI-specific symptom and without any testing.

**Conclusion:**

Inadequate symptom documentation and inappropriate urine testing contribute to a high rate of unnecessary antibiotic prescribing in older female patients with recurrent UTI. Clinical decision support tools that address these gaps could promote antibiotic stewardship.

## Introduction

Over 50% of female patients experience at least one episode of uncomplicated UTI during their lifetime, and 20–30% will have recurrent episodes [1]. Recurrent UTI, defined as at least two infections within 6 months or at least three within a year, affects female individuals of all ages but older women are disproportionately affected with the rate doubling after age 65 [2].

Unnecessary antibiotic prescribing, i.e., antibiotic use for a condition when it is not recommended based on best practices, is a well-known contributor to multi-drug antibiotic resistance, adverse events, and gut dysbiosis that increases risk of future UTIs [3–5]. According to practice guidelines, antibiotics for UTI should be prescribed to female patients with recurrent UTI only when symptoms attributable to UTI (UTI-specific symptoms) are present and urine culture demonstrates the presence of an organism with pathogenic potential [6, 7]. This decision making is challenging in older female patients who have a high rate of asymptomatic bacteriuria, where bacteria with pathogenic potential are present in urine culture in the absence of UTI-specific symptoms, a common scenario for which antibiotics are not recommended [8]. The rate of asymptomatic bacteriuria, already high at 15–20% in older women, increases to 30% in those with recurrent UTI [9]. Additional challenges that clinicians face when prescribing antibiotics to older female patients include differentiating symptoms of UTI from other common co-existing conditions such as overactive bladder and genitourinary syndrome of menopause as well as pressure from patients and caregivers to prescribe antibiotics when only nonspecific symptoms such as fatigue and memory changes are present and which should not be attributed to UTI [6, 10–12]. In these clinical scenarios, an antibiotic for UTI may be prescribed in the presence of documented negative urine culture, absent pyuria, or even without any urine tests. Most antibiotic stewardship programs have focused on hospitalized patients [13]; however, the majority of antibiotics for UTI are prescribed in outpatient visits to physicians’ office and emergency rooms [14]. Consequently, there is a lack of data on the rate of unnecessary antibiotics and the clinical scenarios in which they are prescribed to older female patients with recurrent UTI in the ambulatory setting.

In recent years, antibiotic overuse and multi-drug-resistant UTI are increasingly being linked to non-medical factors that influence health outcomes, i.e., social determinants of health (SDOH). Prior studies have linked race, ethnicity, census-area level deprivation, Medicaid as primary insurance, and need for interpreter to a higher rate of multi-drug resistant UTI [15]. Individuals at socioeconomic disadvantage and those living in rural areas receive more unnecessary antibiotic prescriptions for UTI [16]. However, little is known about how SDOH affect antibiotic prescribing in older female patients with recurrent UTI.

The primary aim of this study was to describe the rate of unnecessary antibiotic prescriptions across specialties in ambulatory clinical practice and the frequency of the clinical scenarios in which unnecessary antibiotics were prescribed in older female patients with recurrent UTI. Secondarily, we sought to describe factors including demographic variables and SDOH that may be associated with unnecessary antibiotic prescriptions.

## Material and Methods

This was a retrospective cohort study of older female patients with recurrent UTI seen in ambulatory practices of a large academic health system. Inclusion criteria were age 65 years and older, and clinical diagnosis of recurrent UTI defined as an antibiotic prescription for a UTI two or more times in the past 6 months or three or more times in the past year [17]. We purposefully chose this treatment-based definition over culture-based definition of recurrent UTI to avoid excluding those with greater social vulnerability who may have limited access to a lab for urine culture. Exclusion criteria were chronic catheter use, a history of renal transplantation, immunosuppressive drugs, anatomic or functional anomaly of the urinary tract system, active treatment for bladder cancer, and UTI episodes in which urine test data were not available.

To identify female patients with a clinical diagnosis of recurrent UTI, an initial electronic health record (EHR) search query was performed using the diagnosis codes N39.0 (urinary tract infection, site not specified) and Z87.440 (personal history of UTI) in women age 65 and older between June 30, 2022 and December 28, 2022. The date when the diagnosis of recurrent UTI had been recorded in the electronic health record by a clinician was identified. Encounters in the 12-month period prior to and following the diagnosis were manually reviewed. A clinical diagnosis of recurrent UTI was made if (i) a clinician had recorded a diagnosis of recurrent UTI in the medical record and (ii) if at least two antibiotic prescriptions for UTI had been prescribed in 6 months or at least three in 1 year. Urine test data were extracted from actual laboratory results available in the EHR. If a test was noted to have been performed outside of the health system but results were not visible within the EHR, the encounter was excluded.

Demographic, symptoms, and antibiotic data were extracted for up to three UTI episodes closest to the time of diagnosis of recurrent UTI. Demographic data included age at time of diagnosis, race, ethnicity, body mass index (BMI), primary insurance, caregiver involvement in telephone/electronic messages, and concurrent diagnoses.

Symptoms were classified as UTI-specific symptoms specifically referrable to UTI (such as dysuria, frequency, urgency, urinary odor, worsening incontinence, suprapubic/lower abdominal pain, hematuria, fever, flank pain) or as nonspecific symptoms often attributed to UTI in clinical practice (such as fatigue, mental changes, back pain not documented as flank pain, and other poorly characterized symptoms such as “patient currently having symptoms”) [6, 8, 18].

Data on standard urine culture performed using standard laboratory practices in the United States were extracted. Positive urine culture was defined as > 100,000 colony forming units (cfu)/ml for at least one organism. Negative cultures included growth < 100,000 cfu/ml or mixed organisms. Asymptomatic bacteriuria was defined as the presence of bacteria thought to be a pathogen > 10,000 cfu/ml in the absence of the UTI-specific symptoms listed above [8, 19].

An unnecessary antibiotic was defined as an antibiotic prescribed when any one of the following were present:

(i) Asymptomatic bacteriuria,
(ii) Absence of UTI-specific symptoms (irrespective of urine test results),
(iii) Negative urine culture,
(iv) Negative pyuria defined as < 10 white blood cells per high powered field on urine micorscopy [6, 8, 19].

This definition did not include urine dipstick results which have lower negative predictive value for clinical diagnosis of UTI in older women [20]. An empiric antibiotic was defined as an antibiotic prescribed before obtaining results of urine testing and classified as (a) empiric with concurrent urine test order or (b) empiric without concurrent urine test order. Antibiotic resistance was defined as resistance to three or more classes of antibiotics. The specialty of the clinician prescribing the antibiotic was recorded.

SDOH were assessed using the social vulnerability index (SVI), a tool released by the Centers for Disease Control and Prevention (CDC) and the Agency for Toxic Substances and Disease Registry [21]. The tool incorporates four SDOH themes (socioeconomic status, household composition and disability, minority status and language, and housing type and transportation household) to determine the overall social vulnerability of each census tract using data collected by the United States Census Bureau. Street address of residence was used to identify each patient’s census tract. SVI scores range from 0 to 1 with higher scores indicating communities with increased social vulnerability.

### Analysis

The primary outcome was the rate of unnecessary antibiotics. Prior studies on the rate of unnecessary antibiotics for older female patients with recurrent UTI are not available. A convenience sample of 175 older patients with recurrent UTI (that encompassed 454 episodes of antibiotic prescription) was used to describe the rate of unnecessary antibiotics and compare it across specialties. The clinical scenario in which an unnecessary antibiotic was prescribed was classified by presence or absence of UTI-specific symptoms and urinary test characteristics (such as asymptomatic bacteriuria, negative culture, negative pyuria, or no testing). SVI percentile rankings were divided into four quartiles (SVI-Q) based on overall rankings (0–0.25 as 1 st quartile [SVI-Q1] and least vulnerable, and 0.75–1.00 as SVI-Q4 and most vulnerable) [22]. The rate of unnecessary antibiotics across specialties was compared using chi-square test or Fisher’s exact test as appropriate. In this descriptive study, the rate of unnecessary antibiotic between specialties was not compared. Demographic data, SVI, access metrics, and antibiotic data were described using means or medians for continuous variables and proportions for categorical variables. Variables were compared between patients who had ever received an unnecessary antibiotic versus those who had never received an unnecessary antibiotic using parametric *t*-tests, non-parametric Wilcoxon rank sum test, or chi-square test as appropriate. *P* values <.05 were considered significant. STATA version 17 (College Station, TX) was used for analysis.

## Results

The electronic search based on the diagnosis code of recurrent UTI identified 557 eligible patients during the study period. Following manual chart review, 382 patients were excluded for the following reasons: did not meet definition of recurrent UTI (*n* = 265), no visit during the study period (*n* = 100), renal transplant (*n* = 6), cancer or anatomic anomaly of urinary tract (*n* = 6), or indwelling catheter (*n* = 5). Data of 175 patients who contributed a total of 454 encounters during which an antibiotic was prescribed are described in the following analysis.

Median age at the time of recurrent UTI diagnosis was 76 (interquartile range (IQR) (72–85). Most patients identified as White (85%), 6% as Black, 3% as Hispanic, and 9% as other races. All patients had insurance with the majority having Medicare (91%). The most common comorbidities were overactive bladder (34%) and urinary incontinence (32%).

Of the 175 patients, 85 (48.5%) were in the low SVI (lowest social vulnerability), 44 (25%) in the mid-low, 25 (14%) in mid-high, and 21 (12%) in the high (greatest social vulnerability) groups. SVI was associated with race (*p* =.001) and primary language (*p* =.002) with the high SVI group having the highest proportion of non-white patients (62%) and lowest rate of patients whose primary language was English (86%). There were no significant associations of SVI with BMI, insurance, comorbidities, or ever seeing a urology/urogynecology specialist (overall rate 30%). Patients in the high SVI (most vulnerable) group had the lowest rate of UTI episodes treated by a primary care clinician (48% vs. 51% vs. 79% vs 60%, *p* =.001), highest rate of ever obtaining care in the emergency department (ED)/urgent care (33% vs 20% vs 5% vs 8%, *p* =.003), and highest rate of caregiver involvement (62% vs 36% vs 29% vs 20%, *p* =.002) (all rates listed as high vs mid-high vs mid-low vs low SVI).

Analysis of the 454 episodes where an antibiotic for UTI was prescribed showed the median number of UTI-specific symptoms documented was 1 (IQR 0–2). No UTI-specific symptom was documented in 111 (24%) episodes. Nonspecific symptoms such as back pain in the absence of documented flank pain (7%), mental changes (e.g., confusion, forgetfulness) (6%), and fatigue (6%) were recorded in 119 (26.2%) episodes. In 17 encounters (3.7%), only nonspecific symptoms were documented.

Urinalysis reports were available for 70% of episodes, urine microscopy for 54%, and urine culture for 81%. Pyuria ≥ 10/hpf was present in 41% (*n* = 186) of episodes. Urine culture was positive for 339 (75% of all episodes) and negative for 30 (7% of all episodes).

Characteristics of the antibiotics prescribed by specialty during 454 episodes of UTI are presented in Table 1. The proportion of episodes treated by specialty included primary care (61.8%), obstetrics and gynecology (9.2%), urogynecology (16%), urology (8%), and ED/urgent care (4.8%). The overall rate of empirical antibiotics with concurrent testing was 51% and differed significantly across specialties ranging from 95% in ED to 34% in obstetrics/gynecology (*p* <.001). The rate of empirical antibiotics without concurrent testing was also significantly different across specialties being highest in primary care (19%, *p* =.03).

**Table 1:** Type of antibiotic prescriptions and clinical scenarios for unnecessary antibiotics across specialties during episodes of UTI in 175 older patients with recurrent UTI

	Overall	Primary care	Ob/gyn	Urogyn	Urology	Emergency department/urgent care	*p* value
	*N* = 454	*N* = 281	*N* = 42	*N* = 73	*N* = 36	*N* = 22	
Empiric antibiotic with urine testing	230 (51)	154 (55)	14 (34)	27 (38)	15 (42)	20 (95)	< 0.001*
Empiric antibiotic without urine testing	67 (15)	52 (19)	4 (10)	4 (6)	6 (17)	1 (5)	0.030*
Overall rate of unnecessary antibiotics (*N*, %)	185 (41)	127 (45)	12 (29)	30 (41)	10 (28)	6 (27)	0.060
Timing of unnecessary antibiotic							0.826
First episode	68 (37)	47 (37)	4 (33)	10 (33)	3 (30)	4 (67)	
Second episode	73 (39)	52 (41)	5 (42)	12 (40)	3 (30)	1 (17)	
Third episode	44 (24)	28 (22)	3 (25)	8 (27)	4 (40)	1 (17)	
Clinical scenarios of unnecessary antibiotic							
No UTI-specific symptoms (irrespective of urine test result)	111 (60)	76 (60)	8 (67)	18 (60)	5 (50)	4 (67)	0.945
Positive culture but no UTI-specific symptoms (asymptomatic bacteriuria)	86 (46)	52 (41)	8 (67)	17 (57)	5 (50)	4 (67)	0.213
White blood cell < 10/high powered field	60 (32)	38 (30)	4 (33)	12 (40)	4 (40)	2 (33)	0.515
Negative culture	30 (18)	23 (22)	1 (8)	3 (10)	2 (20)	1 (17)	0.594
No UTI-specific symptoms and no urine testing	20 (11)	19 (15)	0 (0)	1 (3)	0 (0)	0 (0)	0.087

^*^ *p* <.05

The overall rate of unnecessary antibiotics was 41% and did not differ across specialties. The most common clinical scenario in which unnecessary antibiotics were prescribed was no documentation of UTI-specific symptom (60%). Asymptomatic bacteriuria accounted for 46% of the unnecessary prescriptions. Other scenarios for unnecessary prescription included antibiotics prescribed even when pyuria was absent (32%) or urine culture was negative (18%). In 11% of episodes, an antibiotic was prescribed without documentation of any UTI-specific symptom and without any urine test.

Demographic variables, SVI, and ever seeing a urology/urogynecology specialist were not associated with a patient ever having received an unnecessary antibiotic (Table 2). Patients whose primary language was English were significantly more likely to have received an unnecessary antibiotic (*p* = 0.04).

**Table 2:** Factors associated with recurrent UTI patients who ever received an unnecessary antibiotic

	Overall	Patients who ever received an unnecessary antibiotic	Patients who never received an unnecessary antibiotic	*p* value
	*N* = 175	*N* = 114	*N *= 61	
Age (median, IQR)	76 [71.5–84.5]	74.9 [69.4–83.3]	77.6 [73–84.5]	0.091
BMI (kg/m^2^) (mean, SD)	28.6 (6.4)	28.2 (6.3)	29.3 (6.5)	0.272
Race (*N*, %)				0.784
White	149 (85)	96 (84)	53 (87)	
Black	11 (6)	7 (6)	4 (7)	
Other	15 (9)	11 (10)	4 (7)	
Hispanic/Latino (*N*, %)	6 (3)	3 (3)	3 (5)	0.667
Primary Language English (*N*, %)	172 (98)	114 (100)	58 (95)	0.041
Insurance (*N*, %)				0.605
Private	12 (7)	10 (9)	2 (3)	
Traditional Medicare	90 (51)	56 (49)	34 (56)	
Medicare Advantage	68 (39)	44 (39)	24 (39)	
Low-income Medicare	1 (1)	1 (1)	0 (0)	
Medicaid	2 (1)	2 (2)	0 (0)	
Other	2 (1)	1 (1)	1 (2)	
Social Vulnerability Index (SVI) (N, %)				0.659
Low (< 0.25)	85 (49)	54 (47)	31 (51)	
Mid-low (0.25–0.49)	44 (25)	32 (28)	12 (20)	
Mid-high SVI (0.5–0.74)	25 (14)	15 (13)	10 (16)	
High (>= 0.75)	21 (12)	13 (11)	8 (13)	
Caregiver involvement (ever)	56 (32)	35 (31)	21 (34)	0.615
Ever seen a specialist (urology/urogynecology)	53 (30)	33 (29)	20 (33)	0.598
Comorbidities (N, %)				
Urinary incontinence	56 (32)	38 (33)	18 (30)	0.605
Overactive bladder/urgency incontinence	60 (34)	39 (34)	21 (34)	0.977
Atrophic vaginitis	54 (31)	36 (32)	18 (30)	0.777
Urinary calculus	18 (10)	12 (11)	6 (10)	0.886
Diabetes	43 (25)	28 (25)	15 (25)	0.997
Antibiotic resistance (ever)				0.977
< 3 Antibiotics	103 (69)	66 (69)	37 (69)	
3+ Antibiotics	47 (31)	30 (31)	17 (31)	

There were fewer documented UTI-specific symptoms in episodes of unnecessary antibiotic prescription, with no symptoms documented in 60% of episodes, compared to situations where an antibiotic was prescribed appropriately, with no symptoms documented in only 1% of episodes (Table 3). The number of documented UTI-specific symptoms was also higher when an antibiotic was prescribed appropriately.

**Table 3:** Relationship between number of documented UTI-specific symptoms and unnecessary antibiotics

Number of UTI-specific symptoms	Episode in which an unnecessary antibiotic was prescribed *N* = 185	Episode in which no unnecessary antibiotic was prescribed *N* = 269
0 UTI-specific symptoms	111 (60)	4 (1)
1 UTI-specific symptom	21 (11)	96 (36)
2 UTI-specific symptoms	34 (18)	101 (38)
3 UTI-specific symptoms	19 (10)	68 (25)

## Discussion

The most important finding of this study is that the rate of unnecessary antibiotic prescriptions in older female patients with recurrent UTI is high across specialties. The overall rate of unnecessary antibiotics across 454 episodes of antibiotic prescriptions in 175 older female patients with recurrent UTI was 41% ranging from 45% in primary care to 27% in ED/urgent care. The most common clinical scenario in which an unnecessary antibiotic was prescribed was absence of documented UTI-specific symptoms (60%) followed by asymptomatic bacteriuria (46%). Other clinical scenarios of unnecessary antibiotic prescriptions included absence of pyuria (32%) and presence of documented negative urine culture (18%). In 11% of unnecessary antibiotic prescriptions, antibiotics were prescribed without any documented UTI-specific symptoms and without any testing. These findings suggest that a variety of clinician and patient related factors contribute to a high rate of unnecessary antibiotic prescribing in older female patients with recurrent UTI.

The rate of unnecessary antibiotic in this study is comparable to that of other studies with reported rates of 40–50% in nonrecurrent UTI populations [23, 24]. The most common reason for the unnecessary antibiotic prescription in this study was absence of documented UTI-specific symptoms (60%) when either no UTI-specific symptom or only nonspecific symptoms such as fatigue, mental changes (e.g., confusion, forgetfulness), and back pain were documented. Failure to document UTI-specific symptoms also contributed to the high rate of unnecessary antibiotic prescriptions for asymptomatic bacteriuria. Large prior studies and systematic reviews indicate that mental changes, back pain, and fatigue should not be ascribed to UTI in older adults [20, 25, 26]. Specifically, antibiotic treatment of asymptomatic bacteriuria in older adults with mental changes is associated with a high rate of adverse events, including longer hospital stays, *C. difficile* infection, and mortality [27, 28].

It is possible that UTI-specific symptoms were reported by patients and not documented by clinicians in the medical record. In this study, the number of documented UTI-specific symptoms was higher during episodes in which the antibiotic was considered appropriate based on best practices as compared to those where an unnecessary antibiotic was prescribed. Documenting symptoms encourages clinicians to engage with the decision-making process and provides “accountable” justification for the antibiotic prescription [29]. Documentation of symptoms also allows for tracking and reporting of antibiotics which are core elements of outpatient antibiotic stewardship to measure and modify prescribing practices and align them with evidence-based recommendations. Notably, rates of unnecessary antibiotics for asymptomatic bacteriuria did not differ across specialties, including urology and urogynecology. These findings suggest clinicians across all specialties who take care of older female patients with recurrent UTI would benefit from measures such as documentation templates that facilitate efficient documentation of UTI-specific symptoms and include guidance statements on nonspecific symptoms that should not be attributed to UTI.

Inappropriate ordering and interpretation of urine tests also contributed to the high rate of unnecessary antibiotics. Asymptomatic bacteriuria accounted for half of unnecessary antibiotics indicating that urine tests were ordered in the absence of UTI-specific symptoms and a subsequent result of a positive culture prompted the antibiotic prescription. In 32% of patients who received unnecessary antibiotics, urine microscopy showed the absence of pyuria (white blood cells < 10/hpf). Prior studies have emphasized the importance of “diagnostic stewardship” which involves “modifying the process of ordering, performing, and reporting diagnostic tests to improve the treatment of infections” [30]. For example, clinical decision tools that prompt clinicians to document the reason for ordering the urine test can reduce the number of inappropriate urine tests. Similarly, “reflex” urine cultures where the lab performs a urine culture only if certain predetermined criteria on urine microscopy are met (such as pyuria) can help to reduce the rate of positive cultures and unnecessary antibiotics for asymptomatic bacteriuria.

Patient-related factors likely also contributed to the high rate of unnecessary antibiotics in this study. According to a large database study, the rate of pyelonephritis in older female patients with UTI is only 1.7% [31]; yet the rate of empirical antibiotic prescriptions where antibiotics were prescribed before urine test results were available was high across specialties. Interestingly, the rate of unnecessary antibiotics was higher in patients whose primary language was English and who likely have better access to care than patients whose primary language was not English. Prior qualitative studies show that older patients and their caregivers frequently ascribe nonspecific symptoms such as fatigue and forgetfulness to UTI and have considerable anxiety that these symptoms will progress to “sepsis” [10, 11]. These concerns may have contributed to patients pressuring clinicians to order urine tests for nonspecific symptoms or prescribe antibiotics before urine test results became available. No urine tests were performed in 11% of episodes where an unnecessary antibiotic was prescribed, and 15% of all antibiotics in this study were prescribed empirically without any urine testing. These findings may reflect barriers that prevented access to a lab such as transportation, patient unwillingness to report to a lab, or clinician bias in which patients were recommended testing. These findings suggest that patient education on which symptoms should be attributed to UTI as well as risk of unnecessary urine tests and antibiotics are important elements of antibiotic stewardship in older female patients with recurrent UTI.

Strengths of this study include identification of patients with recurrent UTI through detailed chart review, and stringent definitions of antibiotic outcomes. Limitations include a relatively small sample size. Even though patients in the high SVI group had worse access to care as indicated by highest rate of ever obtaining care in the ED/urgent care, SDOH was not associated with unnecessary antibiotics likely because of the small number of patients in the high SVI group. Additionally, current definitions of recurrent UTI based on culture results or antibiotic prescriptions exclude patients who do not have access to care. We also did not review pharmacist antibiotic prescriptions or measure the dose and type of antibiotics to determine if they were concordant with clinical guidelines. This study was subject to the limitations of the standard urine culture, which may fail to detect bacteria that could cause a symptomatic UTI, thus leading to a false negative result [32]. While we used standard thresholds of a positive urine culture of > 100,000 cfu/ml, this may have reduced the sensitivity of the test and thus may also contribute to a false negative in a patient who is symptomatic [33]. Additionally, while pyuria is a clinically useful benchmark of infection, we acknowledge that microscopy results may have inappropriately reported a negative result due to rapid leukocyte decay in setting of delays from collection to analysis that occur in a real-world setting [34]. Finally, we acknowledge the limitations of the term “asymptomatic bacteriuria” given that emerging studies on urinary microbiome show that bacteriuria should be expected rather than indicative of a disease state [32]. However, this term does have clinical utility given its use in current guidelines as the observed phenomenon of a urine culture that may grow a bacteria of pathogenic potential in the absence of urinary symptoms [8].

In conclusion, the rate of unnecessary antibiotics is high in older female patients with recurrent UTI. This study provides insight into a variety of clinician and patient-related factors, including inadequate symptom documentation and inappropriate urine testing that contribute to this high rate. Clinical decision support tools and patient education and counseling that address these gaps could promote diagnostic and antibiotic stewardship.

## Data Availability

The data that support the findings of this study are available from the corresponding author upon reasonable request.
